# Creating and Using Minimizer Sketches in Computational Genomics

**DOI:** 10.1089/cmb.2023.0094

**Published:** 2023-12-14

**Authors:** Hongyu Zheng, Guillaume Marçais, Carl Kingsford

**Affiliations:** ^1^ Computer Science Department, Princeton University, Princeton, New Jersey, USA.; ^2^ Computational Biology Department, Carnegie Mellon University, Pittsburgh, Pennsylvania, USA.

**Keywords:** de Bruijn graphs, *k*-mer counting, minimizers, read mapping, sketching

## Abstract

Processing large data sets has become an essential part of computational genomics. Greatly increased availability of sequence data from multiple sources has fueled breakthroughs in genomics and related fields but has led to computational challenges processing large sequencing experiments. The minimizer sketch is a popular method for sequence sketching that underlies core steps in computational genomics such as read mapping, sequence assembling, k-mer counting, and more. In most applications, minimizer sketches are constructed using one of few classical approaches. More recently, efforts have been put into building minimizer sketches with desirable properties compared with the classical constructions. In this survey, we review the history of the minimizer sketch, the theories developed around the concept, and the plethora of applications taking advantage of such sketches. We aim to provide the readers a comprehensive picture of the research landscape involving minimizer sketches, in anticipation of better fusion of theory and application in the future.

## 1. INTRODUCTION

Recently, many advances in computational biology have been made possible by the increasing amount data generated from high-throughput sequencing experiments. Processing these sequencing data and extracting biological insights from them efficiently require both improved computing infrastructures and novel algorithms adapted to large-scale data analysis.

Minimizer sketches are a type of “sequence sketch” used to reduce the computational needs of computational biology algorithms. In this context, we use the term sequence sketches to refer to the collection of methods that generate a small representation of a long sequence (See Section 2.2 for more discussion). This small representation is designed to preserve some of the structure and information from the original string. By using these much smaller sketches, it is possible to design algorithms that perform operations between sequences, such as sequence search and sequence alignment, with reduced storage, memory, or computational costs.

### 1.1. Background

The history of minimizer sketches, especially its introduction to computational biology, is worthy of discussion. This is also a story of how big data is playing an increasing important role in computational genomics. The concept of minimizer sketch for computational biology first appears in Roberts et al. (2004a) and Roberts et al. (2004b), and an equivalent algorithm for document fingerprinting, called winnowing, appears in Schleimer et al. (2003), all around the same time. It is first used in computational biology to reduce memory requirements to compute overlaps between sequencing reads: given a large set of sequences, how can one determine which pair of sequences overlap without exhaustively iterating and aligning every possible pair? One solution is to create a mapping from the constituent *k*-mers (substrings of fixed length *k*) to the sequences, and only compare the sequences that have *k*-mers in common. Although this method saves a lot of computation by avoiding aligning many pairs of sequences that do not have any good alignment, it was considered memory prohibitive because of the very large number of *k*-mers to store the mapping (potentially up to $4^{k}$ *k*-mers).

The conceptual idea behind minimizers is to sketch the sequence, or more precisely speaking, to generate a set of *k*-mers for each sequence, a fingerprint, that is much smaller than the original sequence. Applying a minimizer sketch means selecting the minimal *k*-mer in each sliding window of a given sequence (thus the name “minimizer”), and collecting the resulting subset of *k*-mers as the fingerprint (see Section 2 for precise definitions). If the sketch/fingerprint of two sequences has a large overlap, a sequence-level overlap is likely. This holds because if the two sequences have a large overlap, the fingerprint (minimal *k*-mers in sliding windows) on the overlapping part should be shared between the fingerprints.

The idea of minimizer sketching was promising at the time, but one will have to wait for a full decade before minimizer sketches become widely used in computational genomics. There are a multitude of factors behind this development. One such factor is the emergence of next-generation sequencing (NGS), such as those developed by Illumina, that supplanted Sanger sequencing as the dominant sequencing technology. NGS offered shorter reads than Sanger sequencing, but was more accurate, cheaper, and with much higher throughput. The large increase in throughput and reduction of sequencing cost per base lead to a significant increase in data size: typical sequencing depth jumped from ≤10× coverage, to between 50× and 100× or more. This sharp increase in available sequences and large data sets introduced unprecedented computational challenges.

The ever increasing availability of sequencing data from private and public databases, such as the Sequence Read Archive (SRA), the continuing plummeting cost for NGS, and the advent of third-generation sequencing and single-cell sequencing pose even more harsh challenges for computational efficiency. Minimizers and sequence sketching methods have been key instruments in algorithmic developments of computational genomics during the NGS era as they allow great memory and computation reduction with provably little or no cost in accuracy. The methods will likely continue to be a central component in the new generation of core algorithms such as read mapping, sequence search, sequence assembly, and *k*-mer counting. This review presents the theory underpinning minimizers and many of the optimizations and variants proposed over time to improve the original minimizer sketches.

### 1.2. Structure of this review

We provide a comprehensive review of developments in computational genomics around the design, analysis, and application of minimizer sketches. Section 2 provides a formal definition of minimizer sketches, and discusses their properties and relationship to other sequence sketching methods. In Section 3, we focus on the theory side of minimizer sketches, that is, how to design and analyze a minimizer sketch. Contents in this section include various ways to set up a minimizer sketch by choosing its parameters and the various metrics used to analyze the performance of these sketches.

In Section 4, we look at the applications of minimizer sketches, with focus on three representative scenarios: read mapping (including sequence overlapping), sequence assembly, and an umbrella term that we call *k*-mer dispatching, which covers the use case of minimizer sketches for *k*-mer counting, sequence compression, and more general types of sequence comparison. In Section 5, we discuss extensions of minimizer sketches as well as other newer methods that serve a similar purpose. We conclude with some high-level discussion on where the field is heading next in Section 6.

## 2. BASICS OF MINIMIZER SKETCHES

### 2.1. Definition

A minimizer sketch scheme is defined by three parameters w,k, and O: window length, *k*-mer length, and *k*-mer ordering, respectively. *w* and *k* are two integers, and O is a total ordering over all *k*-mers. We use Σ to denote the alphabet, and σ=|Σ|. For most applications in computational genomics σ=4, and we assume σ remains a small constant even in theoretical analyses. A window of a minimizer sketch scheme is a sequence of length w+k−1, or alternatively, a sequence of *w k*-mers. Given a sequence, the minimizer sketch is generated as follows: for each window in the sequence, select the *k*-mer in the window that is the lowest in the ordering O, breaking ties (when the *k*-mer with the lowest ordering appears multiple times) by preferring the leftmost *k*-mer. The sketch of the sequence is the collection of *k*-mers and associated locations that have been selected in any window. Formally:

**Definition 1** (Minimizer and Windows). A “minimizer sketch scheme” or simply a “minimizer scheme” is characterized by (w,k,O) where *w* and *k* are integers and O is a total order over $\Sigma^{k}$. A “window” is a string of length w+k−1, consisting of exactly *w* overlapping *k*-mers. Given a window as input, the minimizer selector outputs the location of the smallest *k*-mer according to O, breaking ties by preferring the leftmost *k*-mer. The *k*-mer at this location is the “minimizer” of this window. The “minimizer sketch” ${ℳ}_{w,k,O}\left(S\right)$, of a sequence *S* given (w,k,O) is the union of all *k*-mers selected in its constituent overlapping windows with their locations in the string.

Throughout this article, we use the term “minimizer sketch” for both the scheme and the resulting sketch, when the context is clear whether the algorithm or the collection of *k*-mer with locations is of interest.

**FIG. 1.**
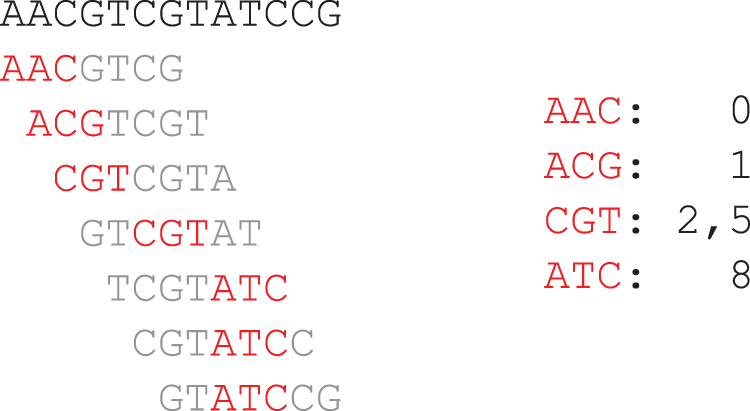
Example minimizer sketch with w=5,k=3 and O being the lexicographical order. Left-hand side: the sequence S=AACGTCGATCCG at the top, each line below is a window of *S* with selected *k*-mer in red. Right-hand side: Resulting sketch of *S*.

Intuitively, minimizer sketches select only a small subset of the *k*-mers in *S*, because adjacent windows likely share the same minimizer. See Figure 1 for an example minimizer sketch with lexicographical order.

#### 2.1.1. Basic properties of minimizer sketches

Minimizer sketches satisfy the following formal guarantees:

**Lemma 1.** Any minimizer sketch satisfies the following three properties:

- (Window) Minimizer sketches are guaranteed to select a *k*-mer at least every *w* bases.
- (Local) If two sequences share a window (have an identical substring of at least w+k−1 bases), it is guaranteed that their minimizer sketch has a common *k*-mer that could be used to locate the matching window in both sequences.
- (Forward) In a sequence, the minimizer picked by any window comes at or after the position of any other minimizer picked in a previous window.

Proof: These properties are direct consequences of the definition.

- Window Property: The minimizer sketch is required to select a *k*-mer in any window, thus it cannot avoid selecting a *k*-mer for at least *w* consecutive bases.
- Local Property: This can be seen by noting that the minimizer selection criteria depend only on the sequence of the window (in particular, the tie-breaking rule is also only dependent on the window itself), and nothing outside the window. Thus, if two sequences share a window, the *k*-mer selected within this window appears in the sketch of both sequences, which can then be used to recover the window match.
- Forward Property: Assume otherwise, that is, there are two adjacent windows (differing by one base) and the *k*-mer *x* selected in the earlier window comes after the *k*-mer *y* selected in the latter window. In this case, *y* is also a *k*-mer in the earlier window, as otherwise *y* would be the last *k*-mer in the latter window and *x* cannot come after *y*. Similarly, *x* is also in the latter window. So in the former window we have x<y (no equality due to tie-breaking rule) and in the latter window we have y<x, leading to a contradiction.

The window property ensures that the sequence is sampled approximately uniformly and that there are no long substrings without minimizer *k*-mers. This property is the basis of applications that use minimizer sketches to break down long sequences in chunks, for example, to parallelize workloads (see Section 4.3). The local property is the most important guarantee for applications such as read mapping, ensuring that long identical substrings (at least as long as a window) between sequences can always be retrieved by comparing their minimizer sketches. Finally, the forward property enables time- and space-efficient implementation of minimizer sketches, by streaming the sequence and capturing minimizer *k*-mers in the stream, as discussed later in this section.

#### 2.1.2. Selecting *w* and *k*

The values of *w* and *k* are of utmost importance for a minimizer sketch. Selection of these parameters is highly dependent on the specific application of the minimizer sketch, and different guidelines exist for different types of applications. For read mapping, the value of *w* presents a trade-off between efficiency and sensitivity: larger *w* means fewer *k*-mers sketched and higher efficiency, but higher potential to miss long matches as only sequences of length w+k−1 are guaranteed to share a *k*-mer. Many read mappers choose a value between 10 and 100, depending on their intended usage (a higher value for lower sequencing error rate and longer sequences, e.g.). The value of *k* is more nuanced and in general should be selected such that *k*-mer collisions are not too frequent. Nowadays, read mappers commonly choose values between 10 and 25.

We discuss read mapping in more detail in Section 4.1. For *k*-mer counting type of application, *w*_0_ and *k*_0_ (to distinguish from the *k* implied by the task) should be selected such that a window is of the same length as the *k*-mers for counting, and *k*_0_ should be selected such that the number of bins ($\sigma^{k_{0}}$ max) yields a reasonable overhead for parallelization. We discuss *k*-mer counting and other related use cases for minimizer sketches in Section 4.3.

#### 2.1.3. Selecting O

As different orderings have no immediate impact on the three important properties of minimizer sketches of Lemma 1, and the order choice does not change the correctness of algorithms using minimizers, the selection of O historically received less attention. The ordering of *k*-mers directly determines which *k*-mers are included in the sketch and which ones are not, so it indeed has a salient impact, for example, on the size of the sketch created. Common ordering choices include the following:

- Lexicographical Ordering: The *k*-mers are ordered by first comparing the leftmost character, then the second in case a tie, and so on. For a DNA alphabet, AAA…A is the smallest *k*-mer and TTT…T is the largest. This is very commonly used due to its simplicity and efficiency in comparing *k*-mers. However, lexicographical ordering has a number of issues, including overrepresentation for stretches of *A*s in the sketch, an overall larger sketch size, and more. These issues were recognized very early on, and remedies of these issue include prioritizing less frequent characters (Roberts et al., 2004a; Roberts et al., 2004b), prioritizing *k*-mers with a certain prefix (Deorowicz et al., 2015), and other tweaks (e.g., see Wood and Salzberg, 2014, on XORing *k*-mers).
- Frequency Ordering. Seen in Chikhi et al. (2015) and commonly adopted in practice, the method assumes existence of a background *k*-mer distribution (e.g., empirical *k*-mer distribution in the latest human reference genome), and the *k*-mers are ordered in a way such that less frequent *k*-mers are given higher priority. Jain et al. (2020) propose a more practical method that demotes (larger in O and thus less likely to be selected) frequent *k*-mers above a certain threshold in a probabilistic way.
- Random Ordering: *k*-mers are ordered randomly for the sketch (although consistent between sequences). A common choice of modern sketches, this has a number of advantages, including less bias toward certain bases, highly efficient (using a hash function), and a small sketch size.

Due to the nature of genomic sequences, minimizers are often defined with “canonical *k*-mers.” The canonical *k*-mer of *m* is either *m* itself or the reverse-complement of *m*, whichever is smaller lexicographically, and the ordering O is defined on canonical *k*-mers only. Using canonical *k*-mers allows, for example, using minimizer sketches on sequencing reads where the sequenced strand is unknown. Although the canonical *k*-mers are routinely used in practice, most of the theory on minimizers, for simplicity reasons, uses normal *k*-mers and ignores the restriction to canonical *k*-mers.

In Section 3, we discuss some other more carefully crafted orderings that improve upon the aforementioned sketches in certain ways.

#### 2.1.4. Implementing a minimizer sketch

Depending on the ordering chosen, implementing a minimizer sketch can be easy or hard. In most scenarios, the ordering comes in the form of (or can be converted to) a hash function, in which case the set of minimizer *k*-mer locations is computable in time linear in the input sequence length (assuming the sequence is sufficiently long, and computing the hash takes constant time) using a monotonic queue. We provide a sample implementation of an algorithm to compute a minimizer sketch. This algorithm has an amortized run time of O(|S|), an improvement over the naive algorithm [whose worst-case run time is O(w|S|)].

The first part is to implement the monotonic queue. This is a data structure that maintains a set *A* of comparable elements, supporting the following operations:

- Insert an element to *A*.
- Remove an element from *A*, in the exact order as they were inserted into *A* (equivalently, remove the current oldest element in *A*).
- Query the current minimum in *A*.

Intuitively, if two elements *x* and *y* are consecutively added to *A* with x>y, *x* can never be the current minimum and can be discarded. Thus, we only need to keep track of elements that are not useless by this rule, which form a queue that is monotonic in both value and time of insertion (thus the name). We use a deque (double-ended queue, allowing adding and removing elements on both ends of the queue) as the underlying data structure.

**Table:** 

***Algorithm 1:** Insert (*X*, *T*) inserts an item to the monotonic queue.*
**input**: Item *X* and time *T*
**1 while** *Rightmost item in deque is larger than X* **do**
**2** Remove rightmost item and time in deque;
**3 end**
**4** Append (X,T) to right end of deque;

**Table:** 

***Algorithm 2:** Fetch (*T*) removes old items and returns the current minimum.*
**input**: Time *T*
**output**: Minimal item X′ with time T′ satisfying T′≥T
**1 while** *Leftmost time in deque is lower than T* **do**
**2** Remove leftmost item and time in deque;
**3 end**
**4** Return leftmost item in deque;

Due to the tie-breaking rule favoring leftmost *k*-mers, the monotonic queue needs to favor item with lower time (i.e., if there are two items with same value but different time, the item with lower time shall be selected). Thus, the item removal rule in Insert reads strictly larger-than. Removal of items from the monotonic queue is performed at query time (Algorithm 2).

In using the monotonic queue, it is necessary that time (the parameter *T*) only increases between calls to Insert and between calls to Fetch. (In some other implementations, the monotonic queue is parameterized by a fixed “window length.” It is equivalent to using our approach but subtracting the window length from *T* during Fetch calls.) If this condition holds, then the items in the queue are in increasing order for both the value of the item and the time of insertion. Based on this time monotonicity condition, we can prove the correctness and efficiency of monotonic queues [explanations are also available in Jain et al. (2020) and Carruthers-Smith (2011) under a different name].

**Lemma 2.** Assume the time monotonicity condition holds. Each time Fetch(*T*) is invoked, the item returned is the minimal item with insert time no less than *T*, assuming such item exists.

Proof: Assume Fetch(*T*) returns $\left(X_{0},T_{0}\right)$, and the correct answer is $\left(X_{1},T_{1}\right)$ instead with $X_{1}<X_{0}$ and $T_{1}\geq T$.

- If $T_{1}>T_{0}$, the item $\left(X_{0},T_{0}\right)$ would have been removed during Insert(*X*_1_, *T*_1_), and cannot be selected during Fetch(*T*).
- If $T_{1}<T_{0}$, the item $\left(X_{1},T_{1}\right)$ would not have been removed during Fetch(T′) for any T′≤T. If the item is removed during Insert($X_{2},T_{2}$) for some item $\left(X_{2},T_{2}\right)$, we have $T_{2}>T_{1}$ and $X_{2}<X_{1}$, so $\left(X_{1},T_{1}\right)$ is not the correct answer. This implies $\left(X_{1},T_{1}\right)$ is still in the deque at the end of Fetch(*T*), and will be selected over $\left(X_{0},T_{0}\right)$ because it is to the left of $\left(X_{0},T_{0}\right)$ in the deque.

The case where $X_{1}=X_{0}$ and $T_{1}<T_{0}$ can be proved in a similar way.

**Lemma 3.** Assume the time monotonicity condition holds. Making *N* calls to the monotonic queue takes O(N) time.

Proof: As the functions have no other loops other than line 2 for both Algorithm 1 and Algorithm 2, it is sufficient to show that these two lines are executed at most *N* times during *N* calls. The only way to add an item to the deque is through line 4 in Algorithm 1, which happens exactly once per Insert call, and thus, at most *N* items would have entered the deque. Line 2 for both Algorithm 1 and Algorithm 2 removes one item from the deque, and thus, they are executed at most *N* times in total.

Lemma 3 implies Insert and Fetch have amortized constant run time.

We now implement a minimizer sketch, assuming that the hash functions are easily calculated. We achieve this by sliding a minimizer window from left to right, and maintaining a monotonic queue with the *k*-mers in the current window.

**Table:** 

**Algorithm 3:** Pseudocode for Implementing a Minimizer Sketch
**input** : Three integers w,k,N and a sequence *S* of length *N*
**output**: The minimal *k*-mer in each of the sliding windows of length *w*,
for a total of N−w−k values
**1 for** i←{0,1,…N−k}
**2** Calculate *X* to be the hash of the $i^{th}$ *k*-mer of *S*;
**3** Call Insert(X,i);
**4 if** i≥(w−1) **then**
**5** Append the *k*-mer represented by Fetch(i−w+1) to output;
**6 end**
**7**

#### 2.1.5. Parallelization

Parallelization of minimizer sketching is also simple, as one can simply divide the sequence into segments and sketch each segment in parallel. However, there are some intricacies when it comes to minimizer *k*-mers at the boundary of segments. A safe method to parallelize minimizer sketch is to split the input sequence into segments with overlaps that are a full window long. This way, minimizer *k*-mers inside the intersection of segments may be counted twice (we can easily deduplicate given the forward property in Lemma 1), but no undercounting is possible.

### 2.2. Relationship to related concepts

#### 2.2.1. Other sequence sketches

Minimizer can be broadly categorized as a sequence sketch in a compressive manner: the size of the resulting sketch is usually smaller than the sequence itself. Methods such as the Burrows-Wheeler Transform (BWT) (Burrows and Wheeler, 1994) and FM-Index (Ferragina and Manzini, 2000) are sometimes also called compressive sequence sketches, but with a critical distinction: minimizer sketches do not aim to preserve the whole sequence but only parts of it, while BWT and FM-Index can be used to recover the full sequence. A common alternative to minimizer sketching is by downsampling *k*-mers, that is, select all *k*-mers that are present in a predefined set. We discuss this line of work in Section 5.2. In addition, for those interested in sequence sketching in general, Rowe (2019) and Marçais et al. (2019) present more thorough reviews for different families of sequence sketching methods.

#### 2.2.2. MinHash

MinHash (Broder, 1997) and minimizer sketches bear some similarity other than their name, but they are also sufficiently different in essence. The positional information of the selected *k*-mers is usually included in a minimizer sketch. Even if a minimizer sketch does not explicitly include the position information, because of the shifting window procedure to create the sketch, the original position of a *k*-mer in the sequence has an effect on whether a *k*-mer is selected. With MinHash, the sketch is created over an unordered collection of elements and there is no notion of position.

An important use case for MinHash is the estimation of distance between sets. Minimizer sketches can be used for distance estimation by discarding positional information, but as discussed in Section 4.4, they are rather ineffective for that specific purpose.

#### 2.2.3. Locality-sensitive hashes

Conceptually speaking, minimizer sketches are also “locality sensitive.” Minor changes in the underlying sequence usually only bring modest changes to the resulting minimizer sketch; if a base in the sequence gets changed, minimizer *k*-mers only change in windows that include the base by the locality as in Lemma 1 (also see Edgar, 2021; Hoang et al., 2022a; Marçais et al., 2018; Shaw and Yu, 2022; for some more discussion on this specific topic). However, there are certain difficulties classifying minimizers directly in the locality-sensitive hash (LSH) framework, as the minimizer sketch consists of *k*-mers alongside their locations, but a typical LSH has a fixed value domain. Moreover, the correctness and performance of algorithms using minimizer sketches are based on the properties given in Lemma 1, not based on bounds of *k*-mer collision probabilities as commonly done in the analysis of LSH.

## 3. THEORIES OF MINIMIZER SKETCHES

In this section, we discuss works on the theoretical front of minimizer sketches. Many theoretical developments of minimizer sketches are tightly connected to (and for many, largely motivated by) applications. With this idea in mind, we organize this section by trying to answer the following questions:

- What is a good minimizer sketch?
- How to build a good minimizer sketch?

Both questions can be discussed in varying contexts: in the asymptotic context (in the limit of large *w* and *k*) and practical ones (tailored for parameter configurations commonly seen in existing applications); in expectation where the sequence *S* is a random sequence; and in the sequence-specific case where the input sequence is fixed (e.g., the human reference genome). Minimizer sketches are also a useful mathematical construct. For example, in samSAMi (Grabowski and Raniszewski, 2013), minimizer sketches are used to build a reduced representation of suffix arrays that allows for efficient searching. However, most theoretical work focuses on improving minimizer sketches themselves.

We organize this section by first discussing theoretical developments around the concept of density (a direct measure of how sparse minimizer sketches pick *k*-mers) in Section 3.1, followed by a discussion of alternative metrics in Section 3.2 and a brief perspective on open problems in Section 3.3.

### 3.1. Density and related techniques

#### 3.1.1. Introduction

Minimizer sketches are used in a very diverse set of contexts. Because of this, there is hardly a unifying measure of “goodness” for a minimizer sketch. Being a sequence sketch, compactness (i.e., relative size) of the sketch naturally comes first as an important metric. In general a compact sketch is considered beneficial as it leads to less data to store in memory and less data to process. There is a long line of work formalizing what it means to be compact, and what makes a compact minimizer sketch, as we describe below.

#### 3.1.2. Definition of density

The density of a minimizer sketch measures, either on average or on a specific sequence, the size of the sequence sketch. Formally speaking:

**Definition 2** (Density). Given a minimizer sketch and a sequence *S*, the “specific density” of the sketch on *S* is defined as $\frac{\left|{ℳ}_{w,k,O}\left(S\right)\right|}{|S|-k+1}$, the number of selected locations in the sketch divided by the number of *k*-mers in *S*. The “expected density” (sometimes abbreviated as density) is the specific density of a sufficiently long random string.

The task of building a good minimizer sketch in this context refers to the task of producing an ordering O given *w* and *k* (and when targeting specific density, also given the sequence *S*) that has a low density.

**FIG. 2.**
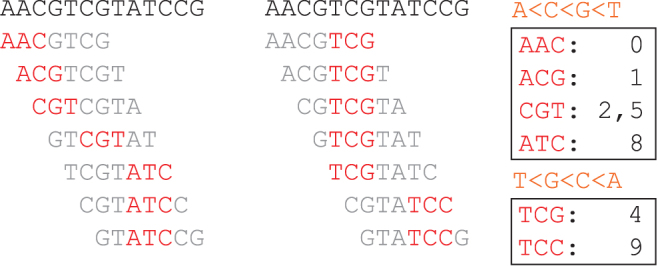
Comparing two minimizer sketches that are identical in w,k and *S*, only differing in the lexicographical ordering O. Left: A<C<G<T, identical to Figure 1. Middle: T<G<C<A and all other parameters intact. Right: Resulting sketches for both setups.

As a concrete example, consider the lexicographical minimizer sketch and sequence *S* as presented in Figure 1. There are 11 *k*-mers in *S*, and 5 of them are present in the sketch, resulting in a specific density of 5∕11. However, as presented in Figure 2, a different choice of O can lead to a very different sketch and the resulting specific density; here, we get a specific density of 2∕11 instead, which is better in this context.

#### 3.1.3. Average case: expected density

Many of the previous studies focus on the expected density, including Schleimer et al. (2003), Zheng et al. (2021b), Zheng et al. (2020), Marçais et al. (2017), and Marçais et al. (2018). Through this line of work, it has been established that the expected density of a random minimizer sketch is 2∕(w+1) under mild conditions (Zheng et al., 2020). For the extreme conditions, when the *k*-mers are much longer than windows (k≫w), the optimal density of 1∕w can be achieved by a carefully constructed minimizer sketch, and when the reverse holds (w≫k), there are no minimizer sketches of density O(1∕w) (Marçais et al., 2018; Zheng et al., 2020).

In addition, when k>w (or when k≈w), a careful construction of the *k*-mer ordering leads to a minimizer sketch of density 1.68∕(w+1) (Zheng et al., 2020). Here, we briefly present a result in this line of work, which involves a number of useful concepts that are also relevant later this section.

**Lemma 4.** When $k>\left(3+U\right)\log_{\sigma }w$ for an arbitrarily small constant of ϵ, a random (w,k)−minimizer sketch has an expected density of 2∕w+o(1∕w).

We provide a proof outline here; see Zheng et al. (2020) for a complete proof. Before the sketch, we need some more technical tools from the classical literature. These can be found in earlier articles (e.g., see Roberts et al., 2004b; Schleimer et al., 2003), and are paraphrased into the presented form in Zheng et al. (2020).

**Definition 3** (Charged context). A “context” of a minimizer sketch is a substring of length (w+k) (equivalently, the union of two adjacent windows). A context becomes a “charged context” for a fixed minimizer sketch, if the minimizer selector selects different *k*-mers in its two constituent windows.

**Lemma 5.** A context is charged for a minimizer sketch (w,k,O), if and only if the minimal *k*-mer in the context (breaking ties by favoring leftmost *k*-mer) is the first or the last *k*-mer.

Proof: If the smallest *k*-mer is neither the first or last one, it belongs to both windows and is the selected minimizer in both windows.

**Lemma 6.** For any sequence *S* and any minimizer sketch (w,k,O) such that *S* is at least w+k bases long, the number of selected *k*-mers in *S* equals the number of charged contexts in *S* plus 1.

Proof: A direct consequence of the Forward property of Lemma 1.

**Corollary 1.** The expected density of a minimizer sketch equals the probability that a random context (a random string of length w+k) is a charged one.

We are now ready to prove Lemma 4.

Proof: A context has w+1 *k*-mers, and the probability that any two *k*-mers in the window are equal to each other is $\sigma^{-k}$. Thus, by union bound, the probability that all *k*-mers are unique is at least 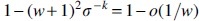. Thus, up to o(1∕w) error, we only need to calculate the probability for a charged context assuming unique *k*-mers. For these contexts, the probability that the context is charged for a random minimizer sketch is exactly 2∕(w+1), as the ordering between all *k*-mers is completely random and there is a 1∕(w+1) chance for each *k*-mer to be minimal.

#### 3.1.4. Universal hitting sets

Another (UHS) method for constructing low-density minimizer sketches revolves around using universal hitting sets, Orenstein et al. (2016). These are set of *k*-mers that must appear at least once in any window. It is straightforward to construct minimizer sketches from a universal hitting set (see Definition 5) with density guarantees, and consequently, building minimizer sketches with low density can be done by building small universal hitting sets as a proxy.

The concept is first proposed in Orenstein et al. (2016), and construction of UHSes has since been refined in multiple ways (DeBlasio et al., 2019; Ekim et al., 2020; Hoang et al., 2022b; Pellow et al., 2017). Here, we highlight some results developed from this line of work, and leave theoretical developments in this front to Section 3.1.2. The core technical tool is the universal hitting set (aka a universal set) and its connection to minimizers:

**Definition 4** (Universal Hitting Sets). A “(w,k)−universal hitting set” *U* is a set of *k*-mers, such that any sequence of *w k*-mers contains at least a *k*-mer from *U*.

In other words, a universal hitting set is a set of unavoidable words: every string containing at least a *w k*-mer “hits” (i.e., intersects) the set.

**Definition 5** (Compatible Minimizer Sketches). Given a (w,k)−universal hitting set *U*, a minimizer sketch (w,k,O) is said to be “compatible” with *U* if and only if O satisfies $x{<}_{O}y$ for any x∈U and y∉U.

**Lemma 7.** Let (w,k,O) be a minimizer sketch compatible with *U*. The expected density of the minimizer sketch is upper bounded by $|U|∕\sigma^{k}$.

Proof: By definition of a universal hitting set, any window of the compatible minimizer sketch contains some *k*-mers from the set. This implies that only *k*-mers in *U* could be selected by the compatible minimizer sketch. Assume the worst-case scenario, that is, whenever a *k*-mer in *U* appears in the sequence, it is selected by the minimizer sketch. In a random sequence, each *k*-mer is also a random *k*-mer and the probability that the *k*-mer belongs to *U* is $|U|∕\sigma^{k}$, so the expected density is exactly $|U|∕\sigma^{k}$ in the worst case.

The importance of compatible minimizer sketches is that it allows us to encode an order on the *k*-mers with desirable properties (as per Lemma 7) by only encoding a set. Encoding a total order on the *k*-mers is more memory consuming: there are $\sigma^{k}!$ possible orders on *k*-mers, taking a superexponential amount of memory to fully encode ($\approx k\sigma^{k}$ bits). In other words, to create an order giving guaranteed low density, it is not necessary to record a total order between the *k*-mers; recording the relative order between a few classes of *k*-mers is sufficient.

#### 3.1.5. Constructing universal hitting sets

A recurring theme in heuristic construction of universal hitting sets is to start from a base set and add *k*-mers as necessary until all sequences of length *L* are covered by a *k*-mer of the set. The *k*-mer to be added to the set is selected using a set of heuristics. We show a simple example.

**Table:** 

**Algorithm 4:** Pseudocode for Finding a UHS
**input**: Integers w,k
**output**: A (w,k)− universal hitting set
**1** Initialize output set *U* with a base set;
**2 while** *U is not a valid* (w,k)− *UHS* **do**
**3 for***Each remaining k*-*mer x*
**4** Count the number of distinct window sequences that contain *x*
but not any *k*-mer in *U*;
**5**
**6** Add the *k*-mer with the largest window count to *U*;
**7 end**

The base set can be the empty set. More commonly the base is a “minimum decycling set” (see Definition 6), that is a minimal size set of *k*-mers that intersects any infinitely long sequences. Starting from a decycling set makes sense as a universal hitting set must also be decycling, and there exists a simple construction for one such set. Then the algorithm extends this set until every path of length *L* is hit.

While useful in practice, the method above has multiple shortcomings. First, there is no guarantee that the generated set is close to optimal. Second, the above algorithm does not scale: the number of *k*-mers grows exponentially with *k*, so the algorithm runs very slowly at even modest values of *k*.

Other heuristics find the longest sequence that is not hit by the set and adds *k*-mers from this sequence to the set. PASHA (Ekim et al., 2020) proposes to parallelize the above algorithm using some approximations, while another method to incrementally expand universal hitting sets with short *k*-mers to universal hitting sets with long *k*-mers is proposed in DeBlasio et al. (2019).

#### 3.1.6. Theory of universal hitting sets

In this section, we take a detour and discuss theoretical development behind the concept of universal hitting sets. This work is tied to the development of minimizer sketches, but many are of independent interest.

For computational complexity of optimizing universal hitting sets, we first define a “sequence-specific hitting set” for a sequence *S* as a set of *k*-mers that intersect with each window of length *w* in *S*. It is known from Orenstein et al. (2016) that finding the minimal sequence-specific hitting set for any sequence *S* of a given length *L* is NP-hard. However, Definition 4 requires the Universal Hitting Set to hit every possible window of a fixed length. This is equivalent to a sequence-specific hitting set for the de Bruijn sequence of order w+k−1 (the window length) with $L=\sigma^{w+k-1}$. It is unknown whether efficient algorithms for sequence-specific hitting sets exist when the input sequence is restricted to de Bruijn sequences.

Besides the hardness in optimization, Universal Hitting Sets are of interest on their own, linking string algorithms (unavaoidable word sets), graph theory via de Bruijn graphs, minimizers, and other sketching methods (see Section 5), through the following concept:

**Definition 6** (Decycling set)**.** A set of *k*-mers *A* is called a decycling set if any sufficiently long string contains a *k*-mer in *A*. In other words, the longest string not containing any *k*-mer in *A* is finitely long.

Decycling sets can also be seen as universal hitting sets with a sufficiently long (but finite) window length. The name “decycling” comes from the fact that these sets are also “decycling” on a de Bruijn graph. Any sequence corresponds to a path in a de Bruijn graph (and conversely), and an infinitely long sequence must contain cycles from the graph. Consequently, a decycling set must intersect every cycle of the de Bruijn graph.

More formally, the following lemma and theorem state the existence and size of minimal decycling sets:

**Lemma 8.** Any decycling set of *k*-mers over an alphabet of size σ must contain at least $N_{\sigma ,k}=\frac{1}{k}\sum_{i|k}\varphi \left(i\right)\sigma^{k∕i}$ *k*-mers, where φ(i) is Euler's totient function. ($N_{\sigma ,k}$ is also known as the number of necklaces or the number of Lyndon words).

Proof: We separate *k*-mers into equivalence classes by rotation, that is, if two *k*-mers *a* and *b* satisfy that a=b[i:k]b[0:i], *a* and *b* are equivalent. The number of equivalence classes is exactly $N_{\sigma ,k}$ (Weisstein, 1995) and each class forms a cycle in the de Bruijn graph. The decycling set must contain one *k*-mer from each equivalence class, as each class corresponds to an infinitely long repeating sequence.

**Theorem 1.** (Mykkeltveit, 1972) For any σ and *k*, there exists a decycling set of size $N_{\sigma ,k}$.

Consequently, minimal decycling sets must be of size $N_{\sigma ,k}$. The proof of Mykkeltveit (1972) is constructive and leads to a practical algorithm to construct one decycling set for each *k*. Champarnaud et al. (2004) gives an alternate construction of a minimum decycling set.

Many relevant questions remain open regarding minimal decycling sets and the link between decycling sets and universal hitting sets. Zheng et al. (2021b) give bounds on the remaining path length of the Mykkeltveit set (the longest string one can write down without hitting a *k*-mer in a Mykkeltveit set), between $\Omega \left(k^{2}\right)$ and $O\left(k^{3}\right)$. Similar bounds for other minimum decycling sets or what range of window is possible with minimum decycling sets is not known.

As discussed above, many of the methods for constructing universal hitting sets take a patchwork approach: start with a minimal decycling set as the base and add *k*-mers to the set until it becomes a universal hitting set for the proper parameter *L*. Interestingly, as recently shown in Pellow et al. (2023), using the Mykkeltveit set as the base set and adding *k*-mers in random ordering (resulting in a universal hitting set of arbitrary size), the resulting compatible minimizer sketch achieves superior density even to sequence-specific methods with prior knowledge of the reference sequence. The surprising result may be attributed to certain structures of minimal decycling sets that are currently unexplored.

The concept of a universal hitting set has also been previously explored in combinatorics, as a special case of nonavoidable words (Bell, 2005; Burstein and Kitaev, 2005; Evdokimov and Kitaev, 2004; Higgins, 2011; Higgins and Saker, 2006; Lothaire and Lothaire, 2002; Saker and Higgins, 2002). Nonavoidable words take a more general definition, where the set contains strings of variable length, including wild cards in several cases. This flexibility also implies that the problem of minimizing the size of a universal hitting set does not have a well-defined equivalent in the world of nonavoidable words. Nevertheless, structural results regarding nonavoidable words would be useful for future research in *k*-mer sets and sketching in general.

#### 3.1.7. Sequence-specific density

A more recent line of work focuses on calculating the density on a specific sequence (DeBlasio et al., 2019; Ekim et al., 2020; Hoang et al., 2022b; Orenstein et al., 2016; Pellow et al., 2017; Zheng et al., 2021a).

##### 3.1.7.1. Universal hitting sets

We have described universal hitting sets in detail in Section 3.1.1. Many of the proposed methods for building universal hitting sets are able to prioritize *k*-mer inclusion from a reference sequence, making them sequence-specific methods. Overall, these methods are able to reduce density by up to 30% (compared with a random minimizer sketch; e.g., see Hoang et al., 2022b) in some practical scenarios on a human reference genome.

##### 3.1.7.2. Polar sets

Polar sets (Zheng et al., 2021a) are another example of construction of an order for low-density minimizer sketches using a set with interesting properties. In a polar set for a sequence *S*, the *k*-mers, similar to polar opposites, repel each other and are guaranteed not to be too close to one another. More precisely:

**Definition 7** (Polar set)**.** Given a sequence *S*, a (w,k)−polar set *A* is a set of *k*-mers such that if the $i^{th}$ and $j^{th}$ *k*-mers of *S* are both in *A*, then |j−i|≥w.

In other words, *k*-mers in the polar set are spread well apart in a sequence, while a universal hitting set makes the opposite guarantee that *k*-mers need to be close together. Compatible minimizer sketches (Definition 5) are similarly defined over polar sets and an analogue of Lemma 7 exists for polar sets, guaranteeing that a well-chosen polar set leads to a minimizer sketch with low density.

Zheng et al. (2021a) relax the definition of polar sets, show that finding a polar set of optimal size is also NP-complete, and give a heuristic algorithm to find polar sets. The heuristic is based on the following idea: if a sequence is not-repetitive and every *k*-mer is unique, it is trivial to find an optimal polar set by taking every $w^{th}$ *k*-mer from *S*. Given a sequence with repeated *k*-mers, the heuristic starts by selecting *k*-mers every $w^{th}$ bases and then updating the set to be a proper polar set.

##### 3.1.7.3. Learning orders

More recently, DeepMinimizer (Hoang et al., 2022b) proposes a more drastic departure from the aforementioned approaches for sequence-specific minimizer sketches. Instead of trying to find a special subset of *k*-mers, a scoring function π over *k*-mers is learned via deep learning. Similar to the case of polar sets, the goal is to create an order that selects, as much as possible, *k*-mers that are *w* bases apart. Because density is not a differentiable objective, it cannot be optimized directly with back-propagation methods. Instead, DeepMinimizer uses two neural networks to optimize a proxy objective, and the total ordering O is generated by comparing scores of *k*-mers.

DeepMinimizer is shown to produce low-density minimizer sketches in some range of parameters (more specifically, large *w* and small *k*) and with very repetitive sequences (e.g., the centromere regions of chromosomes), that previous methods are less effective against.

### 3.2. Other metrics

Density is a straightforward measure for performance of minimizer sketches, but as discussed before, it does not capture every desirable aspect of a minimizer sketch. Several alternative metrics have since been proposed to augment density, and the most interesting candidates are the notions of preservation and balance.

#### 3.2.1. Preservation

Minimizer sketches are known for their robustness: the set of sampled *k*-mers remains relatively stable in the presence of sequence mutations, as we have discussed briefly in Section 2.2. On the contrary, being a *k*-mer-based method (see Section 5 for a discussion of non-*k*-mer-based methods), minimizer sketches are inherently susceptible to mutations because one base change in a *k*-mer results in a totally different *k*-mer, and a base change in a window can change the selected *k*-mer. While many sequencing methods produce high-fidelity sequences with low error rate, there exist sequencing protocols with high error rates (for lower cost, longer sequences, etc.) and mutation rates (especially in cancer samples; see Blanca et al., 2022, for some perspective) that must be taken into account. Therefore, we are interested in the ability of minimizer sketches to preserve *k*-mer matches in the presence of sequence differences.

The concept of *k*-mer preservation comes from multiple applications, including sequence assembly and phylogeny reconstruction. There are no agreed upon definitions of preservation. Here, we present one definition from Hoang et al. (2022a).

**Definition 8** (Preservation)**.** Let *S* be a random string, and S′ be mutated copy of *S*, and (w,k,O) be a fixed minimizer sketch. Let ${ℳ}^{*}={ℳ}_{w,k,O}\left(S\right)\cap {ℳ}_{w,k,O}\left(S\prime \right)$ be the set of shared minimizers between *S* and S′. The “preservation rate” is defined as $\frac{\left|{ℳ}^{*}\right|}{|S|-k+1}$, the number of shared minimizers over the number of *k*-mers in *S*.

Commonly S′ is randomly mutated following a specific distribution, such as i.i.d. mutation per base, although this is not relevant to our discussion here.

**FIG. 3.**
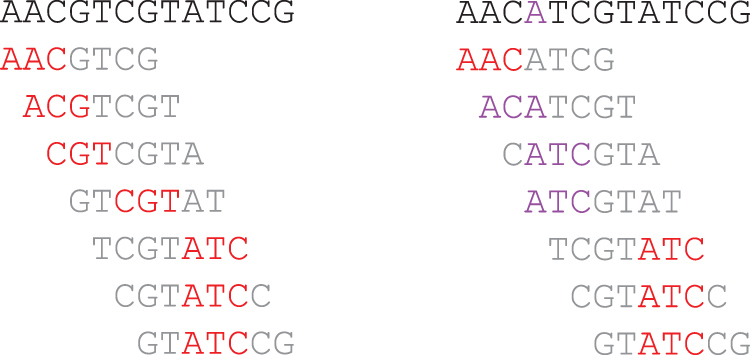
Example for calculating preservation, with setup identical to that in Figure 1. Left-hand side: The original sequence *S*, its windows, and selected minimizer *k*-mers. Right-hand side: The mutated sequence S′ (the single mutated base is marked in purple), its windows, and selected minimizer *k*-mers (those different from *S* are marked in purple). The preservation rate is 2/11.

Figure 3 shows a toy example of calculating preservation of a minimizer sketch. We assume that the only possible mutation of *S* is to change the fourth base to *A*. The preservation rate of the lexicographical minimizer sketch on *S* is 2∕11, as 2 minimizer *k*-mers (out of 5 in *S*) remain in S′, and *S* has a total of 11 *k*-mers. This is also a good example of how seemingly minor changes in the sequence might change the resulting minimizer sketch, in particular when *w* is small compared with *k*: a single base change affects *k* overlapping *k*-mers.

A similar measure of preservation is found in Sahlin (2021) with a different divisor. Edgar (2021), Shaw and Yu (2022), and Dutta et al. (2022) measure preservation by counting the number of bases covered by a common minimizer *k*-mer instead of counting *k*-mers. Frith et al. (2020) focus on shorter sequences and evaluate preservation by whether a pair of sequences with predetermined distance share a minimizer *k*-mer. However, these metrics are highly similar in essence, as they all evaluate the robustness of minimizer sketches against mutations in the sequence.

Unlike existing studies on density and specific density, the studies on building minimizer sketches with improved preservation are largely validated via experiments. In Shaw and Yu (2022), a more tractable formula for preservation is provided and several methods, including some nonminimizer methods, are compared for their preservation metric. This is further extended in Dutta et al. (2022). Hoang et al. (2022a) also evaluate preservation on minimizer sketches and the generalizations, as discussed later in Section 5.

#### 3.2.2. Balance

##### 3.2.2.1. Distance balance

Minimizer sketches are bound by the window property (they must select a *k*-mer every *w* base), so we want the *k*-mers to be selected as sparsely as possible within the limits of window property. One way to quantify the efficiency is the number of *k*-mers picked by a sketch as we have seen in Section 3.1. Alternatively, we can also measure how spread apart the selected *k*-mers are; more spread apart minimizer *k*-mers means more efficient sketches. This balance, or evenness in some contexts, is usually presented by gathering the distance between adjacent minimizer *k*-mers, then plotting the distribution and analyzing its properties such as skewness.

This idea has been mentioned in Edgar (2021) and Frith et al. (2020) [a key construct in Shaw and Yu (2022), the probability vector, is also tightly connected to this concept] and has been qualitatively evaluated, although there are few works that explicitly set out to build such “balanced” minimizer sketches. The polar set method as mentioned in Section 3.1 explicitly enforces a minimum distance between minimizer *k*-mers, but only on some parts of the sequence.

Relatedly, a distinct line of research focuses solely on the idea of maximizing spread between *k*-mers in a mathematically rigorous way. The concept of minimal overlapping words has been previously studied in Blackburn (2015) and Levenshtein (1970), and is connected to computational genomics in Frith et al. (2020). The study by Frith et al. (2023) is a further extension over this line of work, which also proposes a number of different measurements for sequence sketches.

##### 3.2.2.2. Bucket balance

For *k*-mer counting, de Bruijn graph construction, and some other applications (see Section 4.3), minimizer sketches are used to bucket (or rather, partition) *k*-mers such that adjacent *k*-mers are likely to fall within the same bucket. In these scenarios, buckets should not be overwhelmingly large compared with others, and a guarantee that buckets have approximately the same size would also be very helpful. There are several specialized methods for constructing minimizer sketches that specifically aim to improve bucket balance, especially in comparison with lexicographical minimizer sketches known to have bad bucket balance (the homopolymer of *A*s bucket is likely overwhelmingly large, assuming *A* is the lowest character in the ordering). Explicit mentions of this objective can be seen in Nyström-Persson et al. (2021), Ben-Ari et al. (2021), Flomin et al. (2022), Efe (2018), and Marçais et al. (2017).

### 3.3. Open questions

The theoretical development of minimizer sketches and related concepts is far from complete. In this section, we list a number of open directions for future work.

- A nonasymptotic lower bound on density for a minimizer sketch as a function of *k* and *w*. This is especially useful in the case of $k=\Theta \left(\log_{\sigma }w\right)$, which is common in practice and very hard in the sense that current designs are not able to improve performance.
- Similarly, a more refined upper bound of preservation for a minimizer sketch as a function of *k* and *w* in the average case, as well as better designs targeting preservation with performance guarantees.
- As minimizer sketches are used in many different contexts, other measurements of efficiency are desirable. For example, currently, most analyses on preservation of minimizer sketches only focus on mutation by substitution. However, insertion and deletion are also common in genomic data. Thus, a similar measurement of robustness against indels may be of interest. Dutta et al. (2022) and Frith et al. (2023) both propose a number of different measures.
- A more efficient algorithm to design sequence-specific minimizer sketches with lower density (or other related metrics), while remaining efficient to implement, is also wanted. Several existing methods, such as those using an iteratively constructed Universal Hitting Set, require using a lookup table to query membership of a *k*-mer (such as whether the *k*-mer is in the UHS) during sketching. For large value of *k*, the lookup table may become a limiting factor for efficiency, and designs circumventing such limitation may be desirable.
- Due to the nature of genomic sequences, many applications using minimizer sketches only use canonical *k*-mers, defined as the set of *k*-mers that are not larger than its reverse-complement. It is mostly unknown how existing theories regarding minimizer performance apply to canonical minimizers.
- Structures and designs of decycling sets, which underpin many methods in this section and elsewhere (such as open syncmers in Section 5), are also largely unexplored, which may motivate research in sequence sketches in general.
- Lastly, in Section 5, we discuss extensions and alternatives of minimizer sketches. Most of these new concepts are also accompanied by new and exciting theoretical problems regarding their performance.

We believe that interesting lines of research will emerge from these open directions.

## 4. APPLICATIONS OF MINIMIZER SKETCHES

Minimizer sketches are extremely versatile and fit into many major algorithmic building blocks in computational genomics. This section is split into four parts. We first discuss three major categories of using minimizer sketches, and then list other uses that may be less known. Before discussing the methods, it should be noted that many of the tools we discuss in this section are highly complex. While they use minimizer sketches, the use of the sketch might only be a small part of the full picture, and in many cases is a more routine (less novel) component of the method. Thus, we encourage readers to look at individual articles if they are interested in more details.

### 4.1. Read mapping

Minimizer sketches were first proposed for read overlapping (see our discussion in Section 1), and later popularized by their extensive uses in read mapping (among other use cases). While there are some differences, the essential idea of sketching multiple sequences and comparing their *k*-mer contents remains the same. In this section, we discuss this line of work in greater detail. We start with the problem setup, how the minimizer sketches play a natural part in solving the problem, and then discuss the plethora of existing methods that take this route.

#### 4.1.1. The problem

The common setup is as follows (for read mappers): we are given a long reference sequence with preprocessing allowed. Then, a stream of short sequences will arrive, with the expectation that these sequences are approximately subsequences of the reference sequence (differences are common). The objective is to find potential matches against the long reference as fast as possible.

There are many variants to this problem statement, depending on the exact scenario. For example, in RNA-seq, commonly we need to find split matches: that is, the reads (short sequences) might be split into several segments and each needs to be mapped onto the reference to different locations, but the mapped segments should (usually) not be too far away from each other. Resolving differences such as the aforementioned in an efficient way is a key challenge for many modern read mappers. However, the solution usually lies outside the scope of sequence sketches, and so, we do not discuss this in length here.

#### 4.1.2. The recipe

**FIG. 4.**
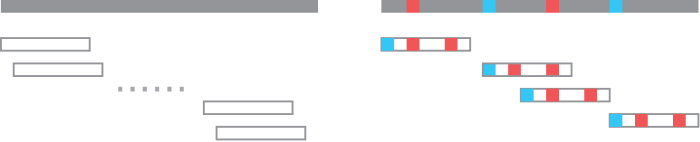
Example of using minimizer sketch for read mapping. Left-hand side presents the original sequence, the original read, and the set of potential mappings. Right-hand side presents their minimizer *k*-mers as colored blocks, and the set of potential mappings that has at least a minimizer match. The second mapping has two minimizer matches, and is usually considered the mapping with highest quality.

Minimizer sketches are a natural fit for read mapping. The common pattern works as follows: we first fix a minimizer sketch (w,k,O) and build the sketch on the reference sequence in preprocessing. Commonly, the reference sketch is indexed using the *k*-mer as key, so given a *k*-mer in the sketch, it is easy to fetch the location of its appearances on the reference sequence. For each incoming read (the short sequences), do the following: using the same minimizer sketch (w,k,O), sketch the read sequence. For each *k*-mer *x* in the read sketch, check if it is also in the reference sketch. Each appearance of *x* in the reference sketch indicates a possible match between reference and read, using the location of *x* as the anchor. Figure 4 provides an example of this approach and shows the potential speedup.

This seed-and-extend approach has been used for a long time, although using the minimizer sketch for the seeding part is a relatively recent invention. Properties of minimizer sketches (see Lemma 1) translate to guarantees over the seeding process. The window property establishes a uniform lower bound of seed coverage on the reference and the read sequences. The local property ensures that credible sequence matches (exact matches as long as minimizer windows) will always be recovered regardless of the sequence and the ordering.

After getting the set of anchor matches, a number of different techniques [direct extension, accelerated anchor chaining (Li and Birol, 2018), etc.] can be used to derive the final mapping, which is out of scope of our discussion.

#### 4.1.3. Examples

##### 4.1.3.1. Mapping to sequences

Minimap (Li, 2016) is one of the most frequently used read mappers that use minimizer sketches with random ordering for quickly finding seed matches. This simple approach has proven to be very powerful and has been adopted by several other works [e.g., see Naznooshsadat et al. (2020); Dilthey et al. (2019); de Sena Brandine and Smith (2021), each with specialization outside the sketching methods]. Minimap2 (Li, 2021; Li and Birol, 2018) improves upon Minimap in various aspects. On the use of minimizers, Minimap2 does not index the original sequence but the HPC (HomoPolymer-Compressed) version of it, with runs of same character collapsed into one single character. It also culls frequent minimizer *k*-mers, which is a common idea present in most of the read mappers using minimizer sketches.

This has been shown to empirically improve the performance. Chromap (Zhang et al., 2021) further builds upon this idea with optimizations to fit the use case of chromatin profiling. MashMap (Jain et al., 2018), an approximate mapper specialized for long reads, uses a hierarchical minimizer sketch (several minimizer sketches with different parameters) to quickly adapt to different read lengths without sacrificing too much time. Winnowmap (Jain et al., 2020) uses a slightly modified version of minimizer sketches called “robust minimizers” (which is discussed in more detail in Section 5) and uses a stochastic two-layer *k*-mer ordering to demote frequent *k*-mers. Notably, it removed the *k*-mer culling step (ignoring the most common *k*-mers in genomes), claiming that step might lead to loss of information.

lra (Ren and Chaisson, 2021) also refines the baseline approach in multiple ways. It filters out minimizer *k*-mers by discarding *k*-mers with high frequency both globally and locally, and a second round of minimizer sketches is used to refine anchor chain matches.

##### 4.1.3.2. Mapping to graphs

The same idea of indexing reference sequences is applicable to indexing sequence graphs. Sequence-to-graph mapping is a young and exciting research area, with many proposed methods centering around the idea of extending seeds obtained from minimizer matches. GraphAligner (Rautiainen and Marschall, 2020) introduces minimizer sketches to sequence graph mapping using a relatively unmodified minimizer sketch: sequences present in the sequence graph are sketched and indexed using minimizers, and their indices are used to find potential seed locations. Pandora (Colquhoun et al., 2021) takes a slightly different route, and directly produces a sketch of the sequence graph using minimizer *k*-mers as nodes of the sequence graph instead; reads are then walked over the “local sketch graph” consisting of minimizer *k*-mers to quickly perform quasimapping.

Giraffe (Sirn et al., 2021), now part of vg toolkit (Garrison et al., 2018), is specialized in matching sequences against gene variation graphs. As the size of the variation graph can be huge and the matches can be ambiguous, Giraffe uses multiple rounds of minimizer seed truncation (discarding repetitive minimizer *k*-mers) and clustering (grouping together minimizer *k*-mers close enough to each other), to reduce the workload for the seed extension part. GED-MAP (Büchler et al., 2023) is a tool for aligning short reads to pan-genome. It constructs the pan-genome reference by first collapsing short variants (such as indels) as wild cards, and then linearizes the remaining graph structure for modeling structural variants. On the linear reference, minimizer *k*-mers can be similarly defined and an algorithm is provided to efficiently find all minimizers, followed by a standard implementation of seed-and-extend for read mapping.

### 4.2. Sequence assembly

Sequence assembly is another important pillar of computational biology, and minimizer sketches also play a role in many modern assemblers. The general task of sequence assembly can be described as follows. Given a set of sequencing reads, the goal is to reconstruct the genome sequence (sometimes multiple sequences considering haplotypes, sometimes a sequence graph) that these reads were sequenced from. There are two (classic) paradigms of sequence assembly: OLC (Overlap-Layout-Consensus) and de Bruijn graph walking. Minimizer sketches are used to facilitate assemblers falling into either category. For example, *k*-mer counting in the sequencing reads is a common first step of assembly pipeline, and minimizers are commonly used for that task (see Section 4.3).

#### 4.2.1. Minimizers in OLC assemblers

For OLC (Overlap-Layout-Consensus) assemblers, minimizer sketches are used to quickly group reads that are likely to overlay each other due to shared minimizers, somewhat similar to how reads are quickly located onto reference sequences in read mapping (Section 4.1). The pioneering articles on minimizer sketches (Roberts et al., 2004a; Roberts et al., 2004b) apply minimizer sketches to quickly perform sequence overlapping. Minimus (Sommer et al., 2007) is another method to directly incorporate minimizer sketches into read overlapping in a rather straightforward manner. In addition to overlapping, ntJoin (Coombe et al., 2020) is a scaffolding tool that constructs a directed graph representing connections between minimizer *k*-mers in assembled segments, and then traverses the graph to quickly sort out orientations and positioning of these segments (usually considered to be part of the layout phase in the OLC paradigm).

While SparseAssembler (Ye et al., 2012) does not directly use minimizer sketches, it uses fixed interval sampling (as discussed later in Section 5), which is a minimizer-like sampling scheme.

#### 4.2.2. Minimizers in other assemblers

Sequence assemblers designed for second-generation short reads typically do not use the OLC paradigm and either use de Bruijn graph-based assembling or hybrid approaches. The construction of the de Bruijn graph from sequencing reads is the central step in this paradigm. Minimizer sketches permit parallelizing this step (see Section 4.3).

LJA (Bankevich et al., 2022) is a new assembler for HiFi reads (Wenger et al., 2019) consisting of multiple novel components, and the first step in the algorithm is to construct a sparse de Bruijn graph over HPC reads (HPC stands for HoloPolymer Compressed as in Minimap2) (Li and Birol, 2018). This is facilitated by using minimizer *k*-mers as vertices with edges denoting adjacency in the originating sequence. Another assembler named WENGAN (Di Genova et al., 2021) is designed for hybrid sequencing libraries, and the first step of the algorithm is to build a de Bruijn graph by assembling short reads, followed by pseudoalignment over synthesized segments from long reads using minimizers in a similar way as Minimap2.

### 4.3. *k*-mer Dispatching

Parallelization is essential to big data analysis, which is increasingly the norm today for computational genomics. As such, parallelization primitives are important for computational genomics, especially those operating over *k*-mers. Minimizer sketches serve as an important tool for parallelization in computational biology, most prominently in *k*-mer counters. In this section, we first discuss how minimizer sketches work in accelerating a *k*-mer counter, and then present how the underlying idea of *k*-mer dispatching finds use in other scenarios.

#### 4.3.1. *k*-mer Counting: dispatching *k*-mers with minimizers

##### 4.3.1.1. Setup and parallelization

Given a sequence *S* and parameter *k*, the task of *k*-mer counting is to produce a table with all *k*-mers in *S* alongside their frequencies. The ordering of *k*-mers in the table is irrelevant as long as each *k*-mer appears at most once. A naive algorithm counting *k*-mers one by one is both slow and memory-intensive. We now present two attempts for parallelization.

- Split *S* into different segments: send each segment to a different process (potentially running on a different machine), perform *k*-mer counting on each segment individually, then collect the tabulation, and merge the counts.
- Split *k*-mers into different buckets: the set of all *k*-mers is partitioned into disjoint sets (by a hash function or a simpler criteria such as the identity of the first three characters), and each process handles counting of *k*-mers in the assigned set. A main process would then stream the sequence *S* and dispatch *k*-mers to the responsible processes. The resulting tabulation is simply concatenated from all processes, because each *k*-mer is counted only in a single process.

However, the first method takes up too much memory during the collection phase (because of merging tables that can be as large as |S|), and the second method takes up too much time during dispatching (each character in the original sequence is sent *k* times to the subprocesses).

##### 4.3.1.2. Parallelization with minimizers

Minimizer sketches provide an elegant approach to parallelize *k*-mer counting, with the advantages of both methods described above. To start, choose a minimizer sketch $\left(w_{0},k_{0},O_{0}\right)$ such that a window of the resulting minimizer is a *k*-mer (formally $w_{0}+k_{0}-1=k$). The method consists of splitting *k*-mers into different buckets, while ensuring that if two *k*-mers have the same minimizer (the *k*_0_-mer selected by the minimizer, by treating the *k*-mer as a window), they belong to the same bucket.

The main process streams the *k*-mers of *S* and dispatches them to the process responsible for counting that *k*-mer. However, because *k*-mers are now bucketed by their minimizers, it is very likely *k*-mers close to each other share the same *k*_0_-mer minimizer, and thus would be sent to the same process. Consecutive *k*-mers that share a *k*_0_-minimizer are merged into a “super-*k*-mer” and a passing super-*k*-mer reduces the communication overhead.

As an example, let k=10, let S[i:j] denote the sequence $s_{i}s_{i+1}\ldots s_{j-2}s_{j-1}$, and assume S[0:10] through S[4:14] all share the same minimizer. Instead of sending these 5 *k*-mers (50 characters), the main process sends S[0:14] (14 characters) to the counting process, reducing communication by 3 times in this specific case. The remainder of the algorithm is mostly the same as the second method, as the final tabulation is done by concatenation.

**FIG. 5.**
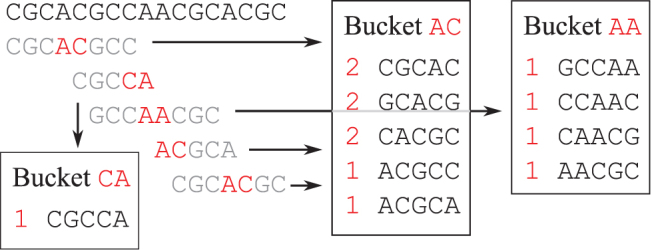
Example of minimizer-assisted *k*-mer counting. For counting 5-mers, we use $k_{0}=2$ and lexicographical order, implying $w_{0}=4$. Left-top shows the sequence *S* and its windows (5-mers) grouped into super-*k*-mers by shared minimizer. Super-*k*-mers are sent to buckets and the results of 5-mer counting are shown in each bucket. The final tabulation is obtained by concatenation.

This saving is typical: as discussed before, the density of a random $\left(w_{0},k_{0}\right)$-minimizer is around $2∕w_{0}$ (see Section 3.1), meaning two minimizer *k*_0_-mers are apart by $w_{0}∕2$ bases on average. For example, using $k_{0}=7$ when counting 21-mers gives $w_{0}=15$ and $w_{0}∕2=7.5$ consecutive *k*-mers are sent at once in average. See Figure 5 for an example of this algorithm; in this specific example, the sequence *S* has 13 5-mers (65 characters), but by using minimizer 2-mers, only 32 characters need to be sent to the buckets.

#### 4.3.2. Examples of *k*-mer dispatching

From the string algorithm viewpoint, the minimizer dispatching method is partitioning *k*-mers into $\sigma^{k_{0}}$ buckets (most commonly for computational genomics σ=4), with the property that *k*-mers that are close to each other on the order-*k* de Bruijn graph are likely to fall within the same bucket, and every instance of a *k*-mer is sent to the same bucket. This property is useful in many applications, and thus, the idea of minimizer dispatching *k*-mers has been independently discovered many times in diverse contexts. Here, we only briefly discuss the use case for each context. Refer to Section 3.2.2 for a discussion on how to build a minimizer sketch specifically for this task.

- *k*-mer counting: We have discussed how this works in the previous section, and the idea is first proposed by MSPKCounter (Li, 2015) and is later followed by a number of improved protocols [see Petrillo et al. (2019); Shibuya et al. (2022); Erbert et al. (2017); Marchet et al. (2020); Mercado et al. (2021) e.g.; they share the same underlying idea, but implement it differently]. KMC2 (Deorowicz et al., 2015) also proposes to exclude *k*-mers fitting certain patterns to improve basket balance, which is carried over to its successor KMC3 (Kokot et al., 2017).
- Building de Bruijn graphs (Ben-Ari et al., 2021; Khan et al., 2022; Li et al., 2013; Marchet et al., 2021; Nyström-Persson et al., 2021; Qiu and Luo, 2017; Rautiainen and Marschall, 2021): Similar to *k*-mer counting, breaking the input sequence into buckets allows building subgraphs in parallel and then reconstructing the de Bruijn graph. Holley and Melsted (2020) operate over colored de Bruijn graphs that support edits, and so, a number of more advanced data structures are needed, however, the core idea remains the same. BCALM and BCALM2 (Chikhi et al., 2016; Chikhi et al., 2015) are methods to generate maximal paths in the de Bruijn graph to compactly store de Bruijn graphs, and these methods rely on careful walking between minimizer bins to ensure correctness. Rengasamy et al. (2018) do not build a full de Bruijn graph, but use a similar idea in one of its steps.
- Sequence compression (Grabowski et al., 2015; Liu and Li, 2021; Liu et al., 2019; Patro and Kingsford, 2015; Wang et al., 2017; Zhang et al., 2020): By dispatching *k*-mers into buckets by minimizers, each bucket is efficiently compressed due to the sequence similarity inside the bucket. In addition, the Bucket Index Correction (BIC) software package (Wang et al., 2017) uses a read error-correction method to find sequences that are similar to each other but differ in minimizer *k*-mers; these differences are treated as “errors” that are corrected at compression and recovered at decompression. In minicom (Liu et al., 2019), the reads in a bucket are further pairwise compared, anchored at the minimizer, and then reordered to achieve maximum compression.
- Caching and indexing: Kraken (Wood and Salzberg, 2014) and Kraken 2 (Wood et al., 2019) are tools to quickly identify metagenomic taxonomy from an underlying *k*-mer database. As the database is huge, it cannot fully reside within memory. The authors propose to organize the database storage by minimizers. When querying the constituent *k*-mers in a string, it is likely that all *k*-mers sharing the same minimizer have been loaded into memory, so no disk read is required if the current *k*-mer shares the same minimizer as the previous one, greatly reducing cache miss rate.
- Kraken 2 further improves upon Kraken by only storing minimizers instead of the full *k*-mers. CONSULT (Rachtman et al., 2021) is a contamination removal tool that contains a coarse *k*-mer lookup table, and the first step is to reduce *k*-mers to their minimizers similar to how Kraken 2 works. Blight (Marchet et al., 2021) implements an exact *k*-mer key-value lookup, and the first step is also to split *k*-mers into minimizer buckets.

### 4.4. Other use cases

We briefly discuss some other interesting use cases of minimizer sketches that do not neatly fit into any of the above categories. A common trait of these use cases is that they are using minimizer sketch as a method to sample *k*-mers from a sequence, and then use the *k*-mer set as a proxy for sequence comparison.

#### 4.4.1. Sequence similarity estimation

As discussed in Section 2.2, minimizer sketches are not LSHs. Nevertheless, minimizer sketches are essentially methods to deterministically sample *k*-mers from a sequence. To estimate sequence similarity, one can use the set of collected minimizer *k*-mers from a string as an estimate of the complete *k*-mer set for that sequence, as seen in Wu et al. (2020) and Li et al. (2020). Furthermore, Jain et al. (2018) propose to estimate sequence identity from potential mappings by using MinHash estimate (as seen in Ondov et al., 2016) over the set of minimizer *k*-mers. This idea is also present in Dilthey et al. (2019). isONclust (Sahlin and Medvedev, 2020) takes a different route where the size of minimizer anchor chain matches is used to estimate the portion of aligned sequences.

However, as pointed out in Belbasi et al. (2022) and also empirically shown in Baharav et al. (2020), this might introduce biases when estimating a simple Jaccard distance. MinMer (Kille et al., 2023) is an extension of minimizer sketches used in MashMap3 achieving significantly reduced bias for Jaccard similarity, which is further discussed in Section 5.1.

#### 4.4.2. Polishing and quality control

Several polishing and quality control methods use minimizers extensively; the set of minimizer matches between sequences is smaller than full *k*-mer matches, but usually representative enough for filtering purposes. HyPo (Kundu et al., 2019) uses well-supported minimizers to split long unpolished sequences into chunks. MiniScrub (LaPierre et al., 2019) extensively uses minimizer *k*-mers as features for learning a quality control model. Minirmd (Liu et al., 2020) uses minimizer matches to detect and deduplicate highly similar reads. WENGAN (Di Genova et al., 2021) uses minimizers to index paired-end sequence collections to polish assemblies from hybrid reads (in addition to using it during the primary assembly).

#### 4.4.3. Sequence membership query

Furthermore, for similar reasons as in Section 4.4.1, minimizer sketches work as efficient samples for large-scale sequence indexing. Raptor (Seiler et al., 2021) uses minimizer *k*-mers as representative samples to quickly match sequences against large sequence databases. Needle (Darvish et al., 2022) further improves upon this idea to perform quantification (counting appearances) of transcript sequences in large collections of experiments by counting minimizer *k*-mers of the transcripts as a proxy. SPUMONI 2 (Ahmed et al., 2023) is a tool for sequence classification, which can be seen as a generalization of membership query against a pangenome. The tool works by representing all sequences using its minimizers and performing classification on the resulting minimizer alphabet. This results in more compact representation and more efficient execution, which is crucial for applications such as nanopore adaptive sampling (Martin et al., 2022) where nontarget reads are discarded during sequencing.

## 5. EXTENSIONS AND ALTERNATIVES

Minimizer sketches are widely useful in many contexts as we have shown in Section 4, but they may not naturally fit into every application. Thus, to develop sketches better suited toward specific needs, many alternatives to minimizer sketches have been proposed. We divide these extensions into three groups. The first two groups are *k*-mer-based and are split by whether or not they still hold the window property (see Definition 1 and Lemma 1). We discuss potential non-*k*-mer -based methods at the end of this section.

### 5.1. Windowed methods

Methods in this section still guarantee that a selection is made in every window.

#### 5.1.1. Local schemes

Local schemes and forward schemes (Marçais et al., 2018; Schleimer et al., 2003) are strict generalizations of minimizer sketches. Local schemes are defined as an arbitrary function mapping a window sequence to a location on the window. Thus, any sketching methods that satisfy the window property are at least a local scheme. Forward schemes are local schemes that satisfy the forward property in Definition 1. Thus, any sketching methods that satisfy all three properties in Lemma 1 are forward schemes. Currently, there is some interest (Marçais et al., 2018; Zheng et al., 2021b) in understanding the design space of local and forward schemes compared with that of minimizer sketches.

#### 5.1.2. Robust winnowing

As mentioned before, minimizer sketches are known for not working well in repeats. Because of the tie-breaking rule favoring leftmost location, any minimizer sketch has to pick almost every *k*-mer in a homopolymer (stretches of the same character). There is a modified tie-breaking rule called Robust Winnowing proposed by Schleimer et al. (2003) and first used by Jain et al. (2020) that avoids such degeneracy. In Jain et al. (2020) and Jain et al. (2022), it has been shown that such methods allow for dropping the common practice of masking high-frequency *k*-mers while being highly effective in speeding up long read mapping. However, from the theoretical perspective, this rule has a side effect: the local guarantee no longer holds as knowing the window content alone is no longer sufficient to determine minimizer picks.

#### 5.1.3. Fixed interval sampling

There is a very simple way to achieve optimal density (Section 3.1) if we do not care about the local guarantee at all: simply select every *w k*-mer in the sequence *S*, which drops the local guarantee as simply inserting a base anywhere before the window changes all the picks after it. Almutairy and Torng (2018) and Khiste and Ilie (2015) use this idea to find maximal exact matches (MEMs) without error between two long sequences. Relatedly, Kutzner et al. (2020) propose a method to extend fixed interval sampling or minimizer sketches to variable-length seeds, including MEMs. These methods are in general greatly constrained as they cannot deal with insertions or deletions at all.

#### 5.1.4. MinMers

MinMers (Kille et al., 2023) is another generalization of minimizer sketches by selecting the minimal *s k*-mers in a window, instead of a single one. MinMers satisfies the window and local property as defined in Lemma 1, but it is not guaranteed to be “forwarding” in the sense a *k*-mer may be selected in two disjoint windows, but not in an in-between window (which is impossible for a regular minimizer sketch). Thus, Algorithm 2 cannot be used for this case, and an efficient algorithm is proposed in Kille et al. (2023) to efficiently construct and query a MinMer sketch using a heap.

This sketch is used for estimation of Jaccard similarity in the same way as described in Section 4.4.1. The MinMer sketch guarantees that the *s* globally minimal *k*-mers are always included, and it is known that a MinHash estimator using *s* smallest hash values has an expected error of $O\left(1∕\sqrt{s}\right)$ [see e.g., Cohen (2014) for more discussion]. Thus, the authors argue that with suitable choice of *s*, MinMer is an asymptotic unbiased estimator for Jaccard distance between sequences. Furthermore, MinMer sketch is also built into a new tool named MashMap3 in the same article for fast approximate sequence mapping.

### 5.2. Nonwindowed methods

Methods in this section no longer guarantee that a selection exists in every window (for many, the entire concept of a window is deprecated), but they still operate based on the concept of *k*-mers.

#### 5.2.1. Direct *k*-mer downsampling

A simple yet elegant way of sampling *k*-mers is to completely ignore contexts and select solely based on the *k*-mer itself. More precisely, a priority set of *k*-mer is generated first (randomly in most cases). A *k*-mer is selected if it is in the priority set regardless of the context (as opposed to a minimizer sketch; the window length *w* is obsolete). This idea has been proposed several times under different names (Dutta et al., 2022; Edgar, 2021; Ekim et al., 2021; Wood et al., 2019).

Using downsampled *k*-mers as nodes (called Universal Minimizers) in a greatly simplified assembly graph, mdbg (Ekim et al., 2021) achieves impressive speedup in sequence assembly. Branchwater (Irber et al., 2022) uses the same technique under the name FracMinHash, and the resulting sketch serves as indexes in sourmash (Brown and Irber, 2016) for large-scale, massively parallel search of petabyte-scale sequence collections. Kraken 2 (Wood et al., 2019) and MashMap3 (Dutta et al., 2022) also include an optional downsampling over a minimizer sketch to further reduce memory usage.

#### 5.2.2. Open syncmers

Open syncmers (Edgar, 2021) are a new class of *k*-mer selection methods that operate in a somewhat similar manner to the Miniception algorithm (Zheng et al., 2020), with an additional parameter *t*.

- Construct a (regular) minimizer sketch $\left(w_{0},k_{0},O_{0}\right)$ whose windows are *k*-mers.
- For a given *k*-mer, apply the minimizer selector on the *k*-mer as a window.
- If the minimizer selects the $t^{th}$ $k_{0}-$mer, the whole *k*-mer is selected in the open syncmer scheme.

It can be seen as an instance of direct *k*-mer downsampling as described in the last paragraph, where no sequence contexts are considered in selecting a *k*-mer. Follow-up works show that open syncmer has great potential in areas such as read mapping and taxonomy classification (Dutta et al., 2022; Sahlin, 2022; Shaw and Yu, 2022). Open syncmers also do not satisfy the local guarantee, and when t>1, open syncmers also naturally avoid oversampling in low-complexity regions. In fact, Edgar (2021) strongly argued against the local guarantee as a requirement for sequence sketches, suggesting that such guarantee provides no protection against sequence mutations.

#### 5.2.3. Masked minimizers/parameterized syncmers

Masked minimizers, proposed in Hoang et al. (2022a), and parameterized syncmer schemes (PSS), proposed in Dutta et al. (2022), are a generalization of both minimizers and open syncmers.

Observe that if an open syncmer with parameters $\left(w_{0},k_{0},O_{0},t\right)$ selects a *k*-mer, the $t^{th}$ *k*_0_-mer was selected by a minimizer with parameters $\left(w_{0},k_{0},O_{0}\right)$. In other words, if we treat open syncmers as methods to select *k*_0_-mers, it is similar to a minimizer sketch followed by a very specific selection process as follows:

- Construct a (regular) minimizer sketch $\left(w_{0},k_{0},O_{0}\right)$ whose windows are *k*-mers.
- Collect all *k*_0_-mers from the above minimizer sketch.
- A *k*_0_-mer *x* is selected if and only if the (unique) window with *x* as its $t^{th}$ *k*_0_-mer has *x* as its minimizer.

Masked minimizers introduce a mask parameter $\nu \in {\left\{0,1\right\}}^{w}$ that replaces *t* in open syncmers, and generalizes the open syncmer selection process as follows:

- The first two steps are identical to the previous description.
- A *k*_0_-mer *x* is selected if and only if there exists some index t∈[0,w−1] with ν[t]=1 such that the window with *x* as its $t^{th}$ *k*_0_-mer has *x* as its minimizer.

In Dutta et al. (2022), a set of locations $S=\left\{x_{i}\right\}\subseteq \left[0,w-1\right]$ play the same role as ν. An open syncmer is simply a masked minimizer with ν being a one-hot vector (equivalently a parameterized syncmer with *S* containing a single element), and a (regular) minimizer is a masked minimizer with ν being a vector of all ones. As masked minimizers are generalizations of open syncmers, they also may not satisfy the window guarantee depending on the mask ν. Dutta et al. (2022) propose to modify the parameterized syncmer sketches by forcing a minimizer selection when a window contains no selected *k*-mers, thus recovering the window guarantee at the expense of selecting more *k*-mers.

Relatedly, Hoang et al. (2022a) also propose a metric called generalized sketch score to evaluate masked minimizers. When applied to (regular) minimizers, it reduces to relative conservation (Preservation in Definition 8 divided by Specific Density in Definition 1). It is shown that certain constructions of masked minimizers improve the generalized sketch score compared with open syncmers and (regular) minimizers. Dutta et al. (2022) propose to evaluate parameterized syncmers using a variety of performance measurements from preservation to percentile of gaps length in sketches, and present tractable formula for several measurements assuming random sequence and random substitution. These measurements are also used to perform hyperparameter optimization for parameterized syncmers and integrate into existing mappers [minimap2 (Li and Birol, 2018) and Winnowmap2 (Jain et al., 2022)], with downsampling and canonicalization (as briefly discussed in Section 3.3). Improvement in long-read mapping performance is observed via experiment on simulated and real data.

### 5.3. Non-*k*-mer-based methods

All methods discussed up to this point select *k*-mers from an input sequence. There are also a multitude of methods that aim to replace *k*-mers, including *k*-mers with wild cards (Binda et al., 2015; Ning et al., 2001; Wood et al., 2019), bidirectional anchors (Loukides and Pissis, 2021), and more classical ones such as variable length matches (related to unavoidable words as discussed in Section 3.1). Among these methods, the Strobemer (Sahlin, 2022; Sahlin, 2021) is a particularly interesting candidate because it is essentially built by chaining minimizer *k*-mers together. The expectation is that such a primitive is more robust to mutations than raw *k*-mers or *k*-mers with wild cards as minimizer sketches are already robust to mutations. It remains to be seen if a minimizer sketch over strobemers is possible, and if they can replace *k*-mers in more applications.

## 6. CONCLUSION

We have provided an extensive review of the theory and application of the minimizer sketches, and related methods. Thanks to their extreme versatility and good performance gains, minimizer sketches have found their way into many algorithms and software packages.

Minimizers provide strong guarantees (e.g., the window guarantee). On the one hand, these guarantees help prove the correctness of algorithms using minimizers, on the other hand, they make developing well performing minimizer schemes difficult. The current research trend is to relax these guarantees, either abandon them entirely or have probabilistic guarantees, or to use machine learning methods to optimize minimizer schemes.

We believe minimizer and related sketching methods will continue to enhance bioinformatic pipelines thanks to continuous rigorous theoretical advancements.
